# Supplementary material for: Chemical representation standardization needed to generalize metabolic pathway involvement prediction across the Kyoto Encyclopedia of Genes and Genomes, Reactome, and MetaCyc knowledgebases
Source: bioRxiv. 2025 Apr 8:2025.04.02.646918. Preprint. [Version 1] doi: 10.1101/2025.04.02.646918 (PMC12026579; doi:10.1101/2025.04.02.646918)
Supplement: Supplement 1 [file NIHPP2025.04.02.646918v1-supplement-1.pdf]

bioRxiv preprint doi: https://doi.org/10.1101/2025.04.02.646918; this version posted April 8, 2025. The copyright holder for this preprint (which was not certified by peer review) is the author/funder, who has granted bioRxiv a license to display the preprint in perpetuity. It is made available under aCC-BY 4.0 International license.

Table 1 – MCC of the dataset and model introduced by Baranwal et al after 50 CV iterations.

Mean MCC	Standard deviation
0.7642	0.0137

Table 2 - Number of identical atom color counts between training set compounds and their cross-references when standardizing the data by converting to SMILES format.

	Bond Stereo On	Bond Stereo Off
Atom Stereo On	2066	2675
Atom Stereo Off	6031	7495

Table 3 - CV analysis for all metrics and all combinations of standardization, atom stereo, and bond stereo.

Atom Stereo	Bond Stereo	Standardized	Metric	Mean Score	Median Score	Standard Deviation
atom-stereo	bond-stereo	not-standardized	Accuracy	0.9984	0.9985	0.0001
atom-stereo	bond-stereo	not-standardized	F1 score	0.8696	0.8708	0.0070
atom-stereo	bond-stereo	not-standardized	MCC	0.8725	0.8737	0.0064
atom-stereo	bond-stereo	not-standardized	Precision	0.7975	0.7986	0.0129
atom-stereo	bond-stereo	not-standardized	Recall	0.9562	0.9566	0.0035
atom-stereo	bond-stereo	standardized	Accuracy	0.9989	0.9989	0.0000
atom-stereo	bond-stereo	standardized	F1 score	0.9031	0.9035	0.0036
atom-stereo	bond-stereo	standardized	MCC	0.9036	0.9040	0.0033
atom-stereo	bond-stereo	standardized	Precision	0.8629	0.8636	0.0092
atom-stereo	bond-stereo	standardized	Recall	0.9473	0.9476	0.0039
atom-stereo	no-bond-stereo	standardized	Accuracy	0.9984	0.9985	0.0001
atom-stereo	no-bond-stereo	standardized	F1 score	0.8675	0.8682	0.0070
atom-stereo	no-bond-stereo	standardized	MCC	0.8707	0.8713	0.0063
atom-stereo	no-bond-stereo	standardized	Precision	0.7929	0.7942	0.0139
atom-stereo	no-bond-stereo	standardized	Recall	0.9578	0.9580	0.0038
no-atom-stereo	bond-stereo	standardized	Accuracy	0.9988	0.9988	0.0001
no-atom-stereo	bond-stereo	standardized	F1 score	0.8974	0.8974	0.0051
no-atom-stereo	bond-stereo	standardized	MCC	0.8985	0.8984	0.0047
no-atom-stereo	bond-stereo	standardized	Precision	0.8467	0.8471	0.0120
no-atom-stereo	bond-stereo	standardized	Recall	0.9547	0.9547	0.0042
no-atom-stereo	no-bond-stereo	standardized	Accuracy	0.9986	0.9986	0.0001
no-atom-stereo	no-bond-stereo	standardized	F1 score	0.8817	0.8839	0.0099
no-atom-stereo	no-bond-stereo	standardized	MCC	0.8840	0.8859	0.0089
no-atom-stereo	no-bond-stereo	standardized	Precision	0.8159	0.8196	0.0202
no-atom-stereo	no-bond-stereo	standardized	Recall	0.9596	0.9603	0.0054
